# Feasibility and willingness-to-pay for integrated community-based tuberculosis testing

**DOI:** 10.1186/1471-2334-11-305

**Published:** 2011-11-02

**Authors:** Neela D Goswami, Emily Hecker, David P Holland, Susanna Naggie, Gary M Cox, Ann Mosher, Debbie Turner, Yvonne Torres, Carter Vickery, Marshall A Ahearn, Michela LM Blain, Petra Rasmussen, Jason E Stout

**Affiliations:** 1 Department of Medicine, Duke University Medical Center, Box 102359, Durham, NC 27710, USA; 2 Wake County Health and Human Services, 10 Sunnybrook Rd, Raleigh, NC 27610, USA

## Abstract

**Background:**

Community-based screening for TB, combined with HIV and syphilis testing, faces a number of barriers. One significant barrier is the value that target communities place on such screening.

**Methods:**

Integrated testing for TB, HIV, and syphilis was performed in neighborhoods identified using geographic information systems-based disease mapping. TB testing included skin testing and interferon gamma release assays. Subjects completed a survey describing disease risk factors, healthcare access, healthcare utilization, and willingness to pay for integrated testing.

**Results:**

Behavioral and social risk factors among the 113 subjects were prevalent (71% prior incarceration, 27% prior or current crack cocaine use, 35% homelessness), and only 38% had a regular healthcare provider. The initial 24 subjects reported that they would be willing to pay a median $20 (IQR: 0-100) for HIV testing and $10 (IQR: 0-100) for TB testing when the question was asked in an open-ended fashion, but when the question was changed to a multiple-choice format, the next 89 subjects reported that they would pay a median $5 for testing, and 23% reported that they would either not pay anything to get tested or would need to be paid $5 to get tested for TB, HIV, or syphilis. Among persons who received tuberculin skin testing, only 14/78 (18%) participants returned to have their skin tests read. Only 14/109 (13%) persons who underwent HIV testing returned to receive their HIV results.

**Conclusion:**

The relatively high-risk persons screened in this community outreach study placed low value on testing. Reported willingness to pay for such testing, while low, likely overestimated the true willingness to pay. Successful TB, HIV, and syphilis integrated testing programs in high risk populations will likely require one-visit diagnostic testing and incentives.

## Background

While targeted testing of high-risk groups for latent tuberculosis infection (LTBI) has long been a priority set forth by the Centers for Disease Control and Prevention [1], LTBI testing has not traditionally been part of community-based HIV outreach programs [2]. Several barriers to community-based integrated screenings incorporating tuberculosis (TB), human immunodeficiency virus (HIV), and sexually transmitted diseases (STDs) exist. Aside from administrative separation of TB, HIV and STD control programs in state and county health departments, perhaps the most significant challenge to incorporating TB testing has been the two-visit process of the tuberculin skin test (TST), requiring participants to return to the community site for interpretation [3]. Follow-up rates for TST have been reported as low as 33% in studies of TB community outreach initiatives [3-5] and have even been problematic when a "mandatory" approach is used: A study in Oregon restricted access to shelters, soup kitchens, detoxification programs, and transitional housing and programs based on participation in TB screening, but still suggested that homeless persons shifting their place of sleep posed a barrier to follow-up [6].

Another barrier is the cost of screening. Many of the persons at highest risk for TB, HIV, and STDs are socioeconomically disadvantaged and either do not have adequate resources to pay for screening or choose not to use their limited resources for such screening. In a survey of 248 symptomatic persons with active tuberculosis in Los Angeles, delay in seeking care was associated with concern about cost [7]. Concern about cost has similarly been demonstrated as a barrier to HIV testing [8].

Furthermore, persons in low-TB incidence settings often have limited knowledge regarding TB, particularly regarding risk reduction measures [9,10]. However, populations targeted for HIV screening do express interest in tuberculosis screening. In a survey of 292 homeless shelter residents in San Francisco, subjects were generally knowledgeable about TB, 57.8% expressed concern about contracting TB infection, and 47.1% described willingness to take measures to avoid TB transmission [11]. We aimed to characterize community acceptance and willingness to pay for and participate in LTBI screening as part of a larger geographic information systems (GIS)-based community outreach integrated screening study.

## Methods

In the context of a larger GIS-based study, residences of individuals diagnosed with TB disease, HIV, and syphilis 1/1/05-12/31/07 in Wake County, North Carolina were mapped using ArcMap 9.3 GIS software, with the areas of highest densities of all three diseases (defined as areas with greater than ten cases per square mile over the three-year period) designated as "hot spots." A map of Wake County streets was overlaid on the study map to determine which street intersections and local businesses were within the "hot spots." In collaboration with nurses and disease intervention specialists from the HIV, syphilis, and TB clinics at Wake County Human Services in North Carolina, we identified sites for local screenings. Sites were chosen based on location within a "hot spot," availability of an area within the site to administer confidential surveys, and acceptability by the site owner/manager. Community-based advertising and small incentives (snacks, beverage and a $5 grocery gift card) were used to attract participants. At each screening event, written informed consent was obtained from each participant. Participants were asked questions regarding medical and epidemiologic TB risk, behaviors, willingness to pay, and health care utilization. After the interview, participants received HIV, syphilis, and LTBI testing. Participants were asked to come to the health department evening clinic for HIV results, and in cases of positive HIV or syphilis results, participants were also contacted directly by a county disease intervention specialist.

In the initial group of patients enrolled in the study (N = 78), two tests for LTBI were performed. Blood was drawn for the Quantiferon Gold In-Tube (QFT-GIT) (Cellestis Limited, Carnegie, Victoria, Australia) assay, an interferon gamma release assay (IGRA) for latent TB. A TST was subsequently placed by the Mantoux method, and persons were asked to return to the same community site or the health department TB Clinic in 48-72 hours for TST interpretation. After an interim analysis (N = 24), the written questionnaire was modified for the remainder of participants (N = 89) due to concern about reliability of responses (see Results).

Patient responses were analyzed with descriptive statistics using SAS software (Cary, NC). The study was approved by the Duke University Medical Center Institutional Review Board and the Wake County Human Rights Consumer Affairs/Human Research Committee. QFT-GIT assays were performed according to manufacturer's instructions, and HIV and syphilis tests were performed according to routine health department protocols.

## Results

A total of 113 participants were recruited through eleven community outreach screenings conducted at six different sites, including a church, a night club, several community centers, and an apartment complex. Of the participants, 54% were male, the majority were African American (90%) and U.S. born (92%), and mean age was 41.4 ± 12.6 (Table 1). The study group represented a population at high-risk for LTBI and STDs, with 71% reporting prior incarceration, 27% reporting current or prior cocaine use, and 35% reporting current or prior homelessness. Furthermore, 53% of persons admitted to unprotected sex within the past year and 49% reported a prior STD. Most subjects (62%) did not have a regular healthcare provider.

**Table 1:** Demographics and Behaviors in Participants of an HIV/syphilis/TB screening (N = 113)

Characteristic	N (%)
Male	61 (54.0)

Race/ethnicity	
Black, non-Hispanic	101 (90.2)
White, non-Hispanic	9 (8.0)
Hispanic	2 (1.8)

Mean age (standard deviation)	41.4 (12.6)

Foreign-born	9 (8.0)

Previous treatment for active or latent TB (self-reported)	12 (10.6)

Previous HIV test (self-reported)	69 (61.1)

Frequency of alcohol use	
None	37 (33.3)
< 1 drink/day	25 (22.5)
1-2 drinks/day	15 (13.5)
> 3 drinks/day	22 (19.8)
> 5 drinks on any day (binge)	12 (10.8)

Tobacco Use	
None	18 (16.4)
Former	8 (7.3)
Current	84 (76.4)

Prior incarceration	79 (70.5)

Current or prior intravenous drug use	8 (7.1)

Current or prior crack use	30 (26.5)

Unprotected sex in past year	58 (52.7)

Prior sexually transmitted disease	51 (49.0)

Current or prior homelessness	40 (35.4)

Perceived risk of HIV/syphilis	
Low	81 (71.7)
Medium	17 (15.0)
High	8 (7.1)
Not reported	7 (6.2)

Perceived risk of tuberculosis	
Low	83 (73.5)
Medium	12 (10.6)
High	11 (9.7)
Not reported	7 (6.2)

The initial 24 subjects enrolled stated they would be willing to pay a median of $20 to get tested for HIV and $10 to get tested for TB (Table 2). After a data quality review, the open-ended questions were removed from the questionnaire because some participants were responding with amounts of money beyond a reasonable range. For example, one participant stated he would pay "two million dollars" for TB and HIV testing. When the survey was modified from open-ended questions (Q1, Q2) to multiple choice (Q4) for the remaining 89 participants, 23% said they would not pay any amount for testing.

**Table 2:** Preferences and Willingness to Pay in Participants of an HIV/syphilis/TB screening (N = 113)

Survey Question	Median or N (IQR or %)
Q1. If testing were not being provided for free today, how much would you be willing to pay to know whether you had HIV? (N = 24)	$20 (0-100)

Q2. If testing were not being provided for free today, how much would you be willing to pay to know whether you had syphilis? (N = 24)	$20 (0-50)

Q3. If testing were not being provided for free today, how much would you be willing to pay to know whether you were infected with the TB germ? (N = 24)	$10 (0-100)

Q4. Would you have come today to get screened for TB, HIV, and syphilis if you had to pay the following amounts (mark the highest amount, with -1 referring to giving out $1 gift cards)? (N = 88)	
-5	2 (2.3)
-1	0 (0)
0	18 (20.5)
1	15 (17.1)
5	53 (60.2)

Q5. How do you feel about getting a skin test (having some liquid injected underneath your skin, like what is done for a TB skin test)? (N = 113)	
Don't mind at all	91 (81.3)
Somewhat unpleasant	14 (12.5)
Hate it	7 (6.3)

Q6. How do you feel about having blood drawn? (N = 113)	
Don't mind at all	79 (69.9)
Somewhat unpleasant	19 (16.8)
Hate it	15 (13.3)

Q7. How difficult is it for you to return in 2-3 days to have your skin test read? (N = 110)	
Easy	98 (89.1)
Somewhat hard	8 (7.3)
Hard	4 (3.6)

Q8. Do you prefer a skin test or blood test? (N = 110)	
Skin test	25 (22.7)
Blood test	43 (39.1)
Doesn't matter	42 (38.2)

Q9. How much would you be willing to pay to get the test that you prefer (instead of the other test)? (N = 113)	$5 (0-20)

Q10. Would you give up your snack to get your preferred test? (N = 45)	
Yes	38 (84.4)
No	7 (15.6)

Q11. Skin testing requires a second visit 2-3 days after the first. How much is it worth to you to avoid that second visit? ($) (N = 113)	$0 (0-5)

Q12. How much time does it take for you to get here from where you would normally be on a weekday (work, home, etc.)? (minutes) (N = 113)	5 (2-20)

Q13. How much time does it take for you to get to the health department from where you would normally be on a weekday (work, home, etc.)? (minutes) (N = 113)	25 (15-40)

Q14. Usually, how difficult is it for you to get transportation for medical appointments? (N = 110)	
Easy	66 (60.0)
Somewhat Hard	21 (19.1)
Hard	23 (20.9)

Q15. Are you working outside the home right now? (N = 110)	
Yes	42 (38.2)
No	68 (61.8)

Q16. Do you have children under the age of 18 living with you? (N = 110)	
Yes	21 (19.1)
No	89 (80.9)

Q17. Do you have a regular doctor? (N = 110)	
Yes	42 (38.2)
No	68 (61.8)

Q18. If you do have a regular doctor, when was your last appointment? (N = 42)	
Within the past 3 months	27 (64.3)
3 months to 1 year ago	8 (19.1)
More than 1 year ago	6 (14.3)
Don't remember	1 (2.4)

Q19. Which test do you trust more to give the "right answer"? (N = 107)	
Skin test	10 (9.4)
Blood test	50 (46.7)
Doesn't matter	47 (43.9)

Q20. Would you give up your snack to know your result in a few minutes? (N = 86)	
Yes	78 (90.7)
No	8 (9.3)

At these high-risk community sites, there was also a slight preference for receiving a tuberculosis blood test over skin test (39% vs. 23%), with more persons trusting the blood test to give the "right answer" versus the skin test (47% vs. 9%, p < 0.001). Participants reported they would even pay a median of $5 (interquartile range (IQR): 0,20) to get their preferred TB test (skin test or blood test). When asked whether they would be (theoretically) willing to give up the free study-provided snack in exchange for testing, 38 (84%) participants agreed that they would give up a free snack for their preferred test.

Seventy-eight persons had a TST placed. For the remainder of participants enrolled in the study (N = 35), TST was omitted from the screening procedure due to poor follow-up in the initial group. Of the total 113 participants, there were four phlebotomy failures and five indeterminate QFT-GIT^® ^results, so 109 persons had valid HIV tests obtained and 104/113 (92%) of persons had valid QFT-GIT^® ^results. Of the 109 persons who had blood drawn for HIV testing, 14 (13%) presented to the health department to receive their HIV results. Similarly, while 89% (98/110 who responded to the question) of participants reported that it would be easy to return to the same site in 2-3 days for TST interpretation, only 14/78 (18%) of participants actually returned for an interpretation. Potential barriers to follow-up for the TST interpretation and HIV result notification visits were reported relatively infrequently, with only 38% reporting full-time employment, 19% reporting children at home, and 40% reporting difficulty with transportation. When asked about perceived risk to get sick with TB, 83/106 (78%) thought their risk was low, and 81/106 (76%) thought their risk to get sick with HIV or syphilis was low.

Results for participants' HIV, latent TB, and syphilis tests were not available at the time of this study, as this data was being collected as part of a larger geographic-based integrated screening study. All of the screenings, however, were performed in two identified "hot spots": the first in the northern part of the county with an average case density of all three diseases of 15.7 cases per square mile, and a second in the central part of the county with an average case density of 19.2 cases per square mile. The remainder of the county had an average case density of less than 10 cases per square mile.

## Discussion

As tuberculosis control efforts move closer to elimination in low-incidence countries, LTBI treatment in relatively high-risk but low-healthcare access populations will become more important to achieve reductions in incident tuberculosis. Community-based screenings may be a reasonable strategy to target such populations, but significant challenges must be overcome to make such screenings feasible and cost-effective. Our study highlights two such challenges: 1) the value that participants place on receiving these screening tests and 2) low return rates for tuberculin skin test readings and HIV result notifications. Our subjects, who reported a number of risk factors for TB exposure as well as for progression to TB disease, perceive their risk of TB to be low. While the test results of specific subjects in this study were not available for analysis, the case density of all three diseases (based on public health surveillance) in our participants' neighborhoods was higher than other parts of the county. Participants in this study also reported relatively low healthcare utilization. The survey responses suggesting that healthcare is a relatively low priority in this population were supported by the low return rates for both TST interpretation and discussion of HIV test results.

While almost all participants expressed a commitment to return for TST interpretation on initial surveys, most did not return for interpretation of tests actually placed in the study, with no clear logistical barriers. Therefore, the only way to effectively reach this at-risk population may be through innovative community outreach programs. With participant preference for a blood-based test that would also eliminate a return visit for interpretation, IGRAs would likely be well-utilized in this kind of community-based outreach effort, and feasibility and acceptability of such tests has been demonstrated [12]. Incentivizing community health workers responsible for connecting LTBI patients with health infrastructure, and using non-incentive based strategies to engage patients in care, such as cellular phone text messaging, are other potential ways to improve patient adherence.

Incentives have enjoyed moderate success in increasing adherence to TB therapy in high-risk populations. In a randomized control trial of homeless persons with LTBI, usual care at a TB clinic was compared to community follow-up with a $5 incentive; completion of isoniazid therapy was significantly higher in the monetary incentive arm [13]. In a California study of LTBI treatment in drug users, therapy completion rate was 53% when active outreach was used with a financial incentive of $5 per visit, compared to 4% when active outreach was used alone (p < 0.0001) [14]. Likewise, in a Haitian study of 60 patients with active TB, one group received free medical care alone, while the second group also received financial aid for three months, travel expenses, nutritional supplements, and monthly clinic reminders [15]. By one year of follow-up, all of the patients in the financially-supported group were cured of their TB, compared to only 57% of patients in the control group. Consistent with these studies, our data support a higher likelihood of participation in TB and STD testing offered in the community with a monetary incentive rather than free medical care alone in a county clinic.

## Conclusion

TB elimination in developed countries will require creative interventions in disadvantaged populations who do not access healthcare regularly and may not place a financial priority on preventive care. TB screening efforts in such populations should employ blood-based methodologies that can simultaneously screen for TB, HIV, and syphilis with a monetary incentive to enhance patient participation.

## Competing interests

The authors declare that they have no competing interests.

## Authors' contributions

NG and JS conceived of and designed the study, performed the statistical analysis, and drafted the manuscript. NG, EH, DH, SN, GC, MB, PR, AM, MA, DT, YT and JS participated in development of the survey tool, subject recruitment, and data collection. EH provided study coordination. DT provided all participant follow-up. All authors read and approved the final manuscript.

## Pre-publication history

The pre-publication history for this paper can be accessed here:

http://www.biomedcentral.com/1471-2334/11/305/prepub
